# Lasiokaurin Regulates PLK1 to Induce Breast Cancer Cell G2/M Phase Block and Apoptosis

**DOI:** 10.7150/jca.93621

**Published:** 2024-03-04

**Authors:** Zhengrui Liu, Jia Wang, Siman Xie, Benteng Zhang, Yan Yuan, Huaizi Fu, Hongyun Hao, Li Sun, Shengtao Yuan, Jian Ding, Hong Yu, Mei Yang

**Affiliations:** 1 Jiangsu Key Laboratory of Drug Screening, China Pharmaceutical University, Nanjing, China.; 2 National key laboratory for multi-target natural drugs, China Pharmaceutical University, Nanjing, China.; 3 Chinese Academy of Sciences Shanghai Institute of Materia Medica, Shanghai, China.; 4 Department of Pathology, The Affiliated Taizhou People's Hospital of Nanjing Medical University, Taizhou, Jiangsu, China.

**Keywords:** Lasiokaurin, breast cancer, G2/M phase block, apoptosis, PLK1

## Abstract

**Aim of the study:** To investigate the anti-tumor effects of Lasiokaurin on breast cancer and explore its underlying molecular mechanism.

**Materials and methods:** In this study, MTT assay, plate colony formation assays, soft agar assay, and EdU assay were employed to evaluate the anti-proliferation effects of LAS. Apoptosis and cell cycle distribution were detected by flow cytometry. The molecular mechanism was predicted by performing RNA sequencing and verified by using immunoblotting assays. Breast cancer organiods derived from patient-derived xenografts model and MDA-MB-231 xenograft mouse model were established to assess the effect of LAS.

**Results:** Our study showed that LAS treatment significantly suppressed cell viability of 5 breast cancer cell lines, with the IC_50_ value of approximately 1-5 μM. LAS also inhibitied the clonogenic ability and DNA synthesis of breast cancer cells, Moreover, LAS induced apoptosis and G2/M cell cycle arrest in SK-BR-3 and MDA-MB-231 cells. Notably, transcriptomic analysis predicted the mechanistic involvement of PLK1 in LAS-suppressed breast cancer progression. Our experiment data further verified that LAS reduced PLK1 mRNA and protein expression in breast cancer, accompanied by downregulating CDC25C and AKT phosphorylation. Ultimately, we confirmed that LAS inhibit breast cancer growth via inhibiting PLK1 pathway *in vivo.*

**Conclusions:** Collectively, our findings revealed that LAS inhibits breast cancer progression via regulating PLK1 pathway, which provids scientific evidence for the use of traditional Chinese medicine in cancer therapy.

## Introduction

Cancer has become the most pressing public health concern, with an estimated 19.3 million new cancer cases and 10 million cancer-related deaths worldwide in 2020 1. In particular, the number of breast cancer cases has increased by 2.3 million, surpassing lung cancer to account for 11.4 percent of all new cancers in women. To date, breast cancer still has the highest number of new cancer cases among women, and also becomes the leading cause of morbidity and mortality in a majority of countries 2-4. Currently, there are six main clinical treatment strategies for breast cancer: surgery, radiotherapy, chemotherapy, traditional Chinese medicine (TCM) therapy, biological therapy, targeted therapy, etc^5^. However, the prevalence of individualized differences makes it difficult to estimate the therapeutic effect of the same drug on different people, which could cause patients and their families to suffer unnecessary pain and economic burden 6, 7. Therefore, the future concept of breast cancer therapy aims to achieve the goal of individualization, as well as treatment escalation or de-escalation based on tumor biology and early treatment response 8.

*Isodon rubescens* is a traditional Chinese herbal medicine containing a wide range of bioactive components, including terpenoids, flavonoids, alkaloids, polysaccharides, amino acids, and some others 9-12. It has been proven that possesses anti-tumour 13-15, anti-bacterial 16-18, anti-oxidization 19 and immune-enhancing properties through modern pharmacological research and clinical trials. As one of its main active ingredients, Lasiokaurin (LAS) may have certain anti-tumor effects. Over the decades, the studies of LAS have been conducted about its positive influence against lung adenocarcinoma, hepatocellular carcinoma, gastric carcinoma and histiocytic lymphoma 20-22. However, the anti-tumor effect of LAS *in vivo* and its mechanism are still unclear, which suggests that a more in-depth study of LAS will be essential and promising.

So far, five PLK family members, including PLK1, PLK2, PLK3, PLK4 and PLK5, have been identified in human cells, among which PLK1 is the most studied. PLK1 plays a variety of important functions across the cell cycle, such as regulating centrosome maturation and spindle assembly 23, activating the anaphase-promoting complex/cyclosome (APC/C) 24, mediating mitotic exit and cell division 25, taking part in nuclear envelope rupture 26, and maintaining genome stability 27. Importantly, abnormal progression of the cell cycle is considered a fundamental mechanism of tumorigenesis, and diverse cyclin-dependent kinases (CDKs) participate in this process to regulate the fidelity and integrity of DNA replication, as well as the timely and accurate segregation of sister chromatids 28. Besides, emerging evidence suggests that PLK1 functions in the damage response, autophagy, apoptosis, and cytokine signaling specifically 29-31. Previous studies have reported that blocking PLK1 expression by antibodies, RNA interference (RNAi), or kinase inhibitors can prevent the proliferation of tumor cells and induce apoptosis 32, 33. Therefore, PLK1 is regarded as a promising target for cancer therapy.

Breast cancer poses a non-negligible danger to the health of women worldwide. Thus, it is urgent to find safe and effective treatment drugs. Lasiokaurin (LAS) is a kind of diterpenoid compound which can be extracted and isolated from the genus Ambrosia. The present study was thus conducted to evaluate the cytotoxic efficacy of LAS against human breast cancer. Furthermore, we sought to reveal the potential target and molecular mechanisms involved in the effects of LAS on breast cancer.

## Materials and methods

### Chemicals and reagents

LAS was supplied by Jiangsu Yongjian Pharmaceutical Technology Co., LTD (Jiangsu, China). TAX was purchased from Jiangsu Yangzijiang Pharmaceutical Group Co., LTD (Jiangsu, China; batch number: 22071611). Volasertib was purchased from MedChemExpress (New Jersey, USA). Methyl thiazolyl tetrazolium (MTT) was purchased from Shanghai Biyuntian Biotechnology Co., LTD (Shanghai, China). Cell counting kit-8 was purchased from Nanjing Nuoweizan Biotechnology Co., LTD (Jiangsu, China). Crystal violet was purchased from Nanjing Sunshine Biotechnology Co., LTD (Nanjing, China). Cell Cycle and Apoptosis Analysis Kit was purchased from MedChemExpress (New Jersey, USA). Cell-Light EdU Apollo488 *In vitro* Kit (100T) was purchased from Guangzhou RiboBio Co., Ltd (Guangzhou, China).

### Cell culture

Human breast cancer cell lines SK-BR-3, MDA-MB-231, BT-549, MCF-7 and T-47D were obtained from the Cell Bank of Shanghai Institute for Biological Sciences, University of Chinese Academy of Sciences. SK-BR-3 was cultured in Dulbecco's Modified Eagle Medium/Nutrient Mixture F-12 (DMEM/F-12) (Gibico, Grand Island, USA). BT-549 was cultured in RPMI-1640 medium (Gibico, Grand Island, USA). MDA-MB-231, MCF-7 and T-47D were cultured in Dulbecco's Modified Eagle Medium/Nutrient Mixture F-12 (DMEM/F-12) (Gibico, Grand Island, USA), 10% fetal bovine serum (FBS, Royacel, China) and supplemented with 100 U/mL penicillin, 0.1 mg/mL streptomycin, and 50 μg/mL gentamicin, and incubated in a humidified atmosphere (Thermo, Germany) with 5% CO_2_ at 37°C.

### Organoid Model

The human breast cancer lump (provided by the Affiliated Taizhou People's Hospital of Nanjing Medical University, Taizhou, Jiangsu, China) was cut into 1 mm^3^ small pieces, and the tumor tissue was placed under the kidney capsule of mice, and mice was monitored every day and waited for the tumor formation. Tumor blocks were removed from PDX mice, washed with PBS and cut into 1 mm^3^ pieces, the tumor tissue was placed in a 15 mL centrifuge tube, digested with digestive fluid for 3h, appropriate amount of PBS was added, filtered with 100 μm filter, centrifuged at 800 rpm for 3 min, the medium was cleaned, the supernatant was discarded, and appropriate amount of matrix glue was added for mixing. It was inoculated in 12-well plates, solidified at 37°C, and then added into the breast cancer medium for further culture.

### Cell viability assay

MTT was performed to determine the effect of LAS treatment on the viability of breast cancer cells. Briefly, breast cancer cells were respectively seeded into 96-well plates, 5 × 10^3^ cells per well. After incubation for 12 h, cells were treated with LAS for another 72 h. Then, MTT 20 μL was added into the medium in each well and incubated for another 4 h. The absorbance was detected at 490 nm using a Microplate reader (Tecan, Durham, NC, USA), and the inhibition rate was calculated by the formula: Inhibition rate (%) = (1- absorbance of the treated group / absorbance of the control group) × 100%.

### Colony formation assay and soft agar assay

The colony formation assay was performed in a 6-well plate, 1000 cells were seeded and incubated for 24 h. Subsequently, cells were treated with LAS and other drugs for about 14 days. The cells were fixed with 4% formaldehyde and stained with 1% crystal violet. Colonies were counted by ImageJ software. The soft agar assay was performed in a 12-well plate. These wells were coated with 1.2% soft agar containing different concentrations of LAS or TAX, followed by seeding of the cells in 0.7% soft agar. After the agar has solidified, a medium with different concentrations of BPH or TAX was added. The number of cell spheres was then counted macroscopically.

### Cell cycle and apoptosis analysis

For cell cycle assay, cells were seeded in a 6-well plate at a concentration of 8 × 10^4^cells/well. After incubation for 12 h, cells were treated with LAS or TAX for another 48 h. The distribution of the cell cycle after LAS treatment was stained by propidium iodide (PI) and detected by using FACS-Calibur flow cytometry. For cell *apoptosis* assay, the cells were treated with LAS or TAX for another 48 h after incubation for 12 h. The condition of the apoptosis after LAS treatment was stained by propidium iodide and Annexin V-FITC. The samples were detected by flow cytometry (BD Biosciences, San Jose, SA).

### 5-Ethynyl-2′-deoxyuridine (EdU) assay

For the EdU assay, cells were seeded in a 96-well plate at a concentration of 1500 cells/well. After incubation for 12 h, cells were treated with LAS or TAX for another 72 h. EdU detection kit was used to determine the cell proliferation status of cells according to the manufacturer's instructions.

### Immunoblotting assay

After incubation for 12h in a 6-well plate, cells were treated with LAS, or TAX for another 48 h. Then, these cells were collected with RIPA buffer containing protease and phosphatase inhibitors and let still at 4°C for 10 min to obtain lysates. Cell lysates were centrifuged at 12000 rpm for 15 min at 4°C and the protein concentration of lysates was estimated via the bicinchoninic acid (BCA) assay. The same amount of protein was separated on SDS- polyacrylamide gel electrophoresis and transferred to PVDF mem- branes. The membranes were blocked with 5% skimmed milk in TBST at 37°C for 5 min, and then incubated with primary antibodies overnight at 4°C followed by incubation with secondary antibodies for 2 h at room temperature. Primary antibodies were purchased from Cell Signaling Technologies and all these antibodies were diluted at a ratio of 1:1000 with TBST containing 5% BSA. Goat polyclonal anti- rabbit IgG conjugated to HRP and goat polyclonal anti-mouse IgG con- jugated to HRP (Cell Signaling Technology, MA, USA) were used as secondary antibodies and all these antibodies were diluted at a ratio of 1:5000 with TBST. Enhanced chemiluminescence reagents (Millipore, MA, USA) were used for detection and exposed by Gel (2000) image analyzer (Bio-Rad, CA, USA).

### MDA-MB-231 subcutaneous tumor model

Female Balb/c nude mice (5 weeks) with bodyweight from 18 to 22 g were purchased from Beijing Huafukang Biotechnology (Beijing, China). After adaptive feeding for a week, 1 × 10^6^ MDA-MB-231 cells were injected subcutaneously of armpit. After tumor volume had grown to about 100 mm^3^, mice were divided into four groups. Then, mice in LAS groups start to administrate LAS intraperitoneal injection once two days. TAX was injected intravenously with a concentration of 10 mg/kg twice a week, and the negative group was given an equal amount of physiological saline. After LAS administration for 27 days, mice were euthanized, and the tumor tissues were then resected and detected. Tumor volume (TV) was calculated by the following formula: TV (mm^3^) = 1/2 × A × B^2^(A, the longest diameter of tumor; B, the shortest diameter of tumor). Animal care and surgery operations were all guided by Animal Care and Control Committee in China Pharmaceutical University.

### Statistical analysis

All data in the experiment were expressed as mean ± S.D. Statistical significance was analyzed by Graph Pad Prism 9.0.0 with one-way ANOVA. *p < 0.05, ** p < 0.01, and *** p < 0.001 represent statistical differences.

## Results

### Effects of LAS on the viability of human breast cells

**Figure 1:**
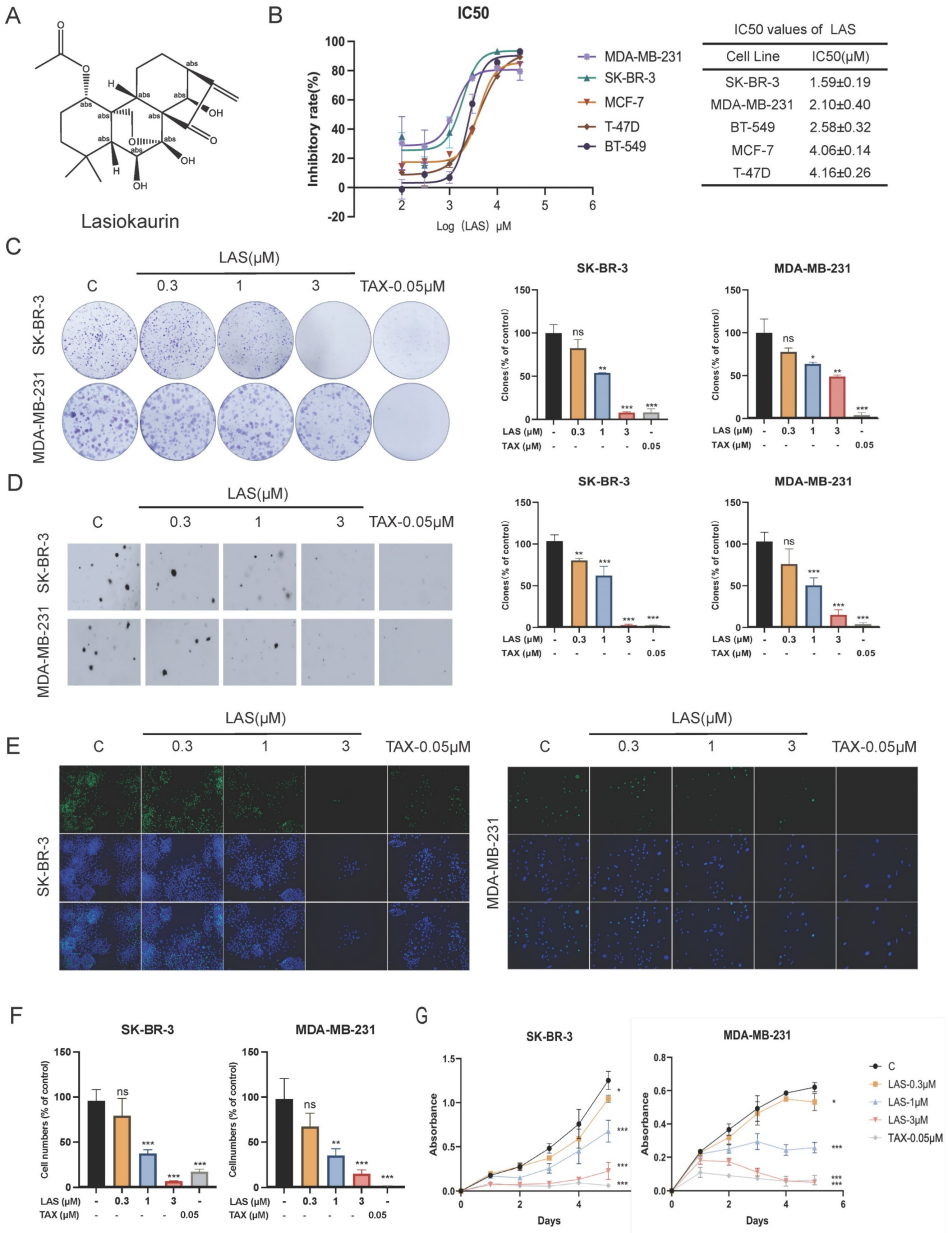
** Effects of LAS on the viability of human breast cancer cells.** (A) Chemical structure of LAS. (B) Human breast cancer cells (SK-BR-3, MDA-MB-231, BT-549, MCF-7 and T-47D) were treated with LAS 72 h, respectively. Cell viability was measured by MTT assay. (C) Clone formation of SK-BR-3 and MDA-MB-231 cells after treatment with indicated concentrations of LAS. (D)Soft agar clone formation of SK-BR-3 and MDA-MB-231 cells after treatment with indicated concentrations of LAS. (E-F) EdU staining assays showed that LAS inhibited DNA synthesis in the SK-BR-3 and MDA-MB-231 cells. Scale bar = 100 μm. (G)Growth curve analysis was conducted to examine the cell proliferation in SK-BR-3 and MDA-MB-231 cells after treatment with indicated concentrations of LAS. Data are represented as the mean ± SD of three independent experiments. The p-values < 0.05 were considered statistically significant for all tests (*p < 0.05, **p< 0.01, ***p < 0.001).

Lasiokaurin (LAS) is a nature diterpenoid derived from *Isodon rubescens*. The chemical structure of LAS was shown in Fig. 1A. To evaluate the anti-tumor effect of LAS against breast cancer, we first measured the anti-proliferative effect of LAS on 5 breast cancer cell lines using MTT cell viability assays. As shown in Fig. 1B, LAS significantly suppressed the cell viability of those 5 BC cells, SK-BR-3, MDA-MB-231, BT-549, MCF-7, T-47D cells, with IC_50_ of approximately 1.59 μM, 2.1 μM, 2.58 μM, 4.06 μM, 4.16 μM, respectively.

Next, we evaluated the anti-proliferative effect of LAS on BC cells using plate clone formation assay. Fig. 1C showed the less and smaller clonal clusters of SK-BR-3 and MDA-MB-231 cells treated with LAS compared to that of controls, suggesting the potent anti-proliferative activity of LAS against BC cells (Fig. 1C). Consistently, data from soft agar colony formation assays showed that LAS can significantly reduce the tumorigenicity of BC cells, with a decreased number of cell colonies seen in SK-BR-3 and MDA-MB-231 cells treated with LAS compared to that of controls (Fig. 1D). Additionally, EdU (5-bromo-2-deoxyuracil), a pyrimidine analogue that can be integrated into DNA double strands during DNA synthesis, was used to detect DNA replication activity of BC cells. As shown in Fig. 1E-F, LAS treatment significantly decreased the incorporation of the fluorescent nucleoside EdU into SK-BR-3 and MDA-MB-231 cells DNA, indicating that LAS could significantly inhibit breast cancer cells proliferation. LAS could significantly inhibit the viability of breast cancer SK-BR-3 and MDA-MB-231 cells in a concentration dependent manner (Fig. 1G). Thus, findings from the tests in those different paradigms all demonstrate that LAS inhibits the growth of breast cancer.

### LAS induced apoptosis of breast cancer cells

**Figure 2:**
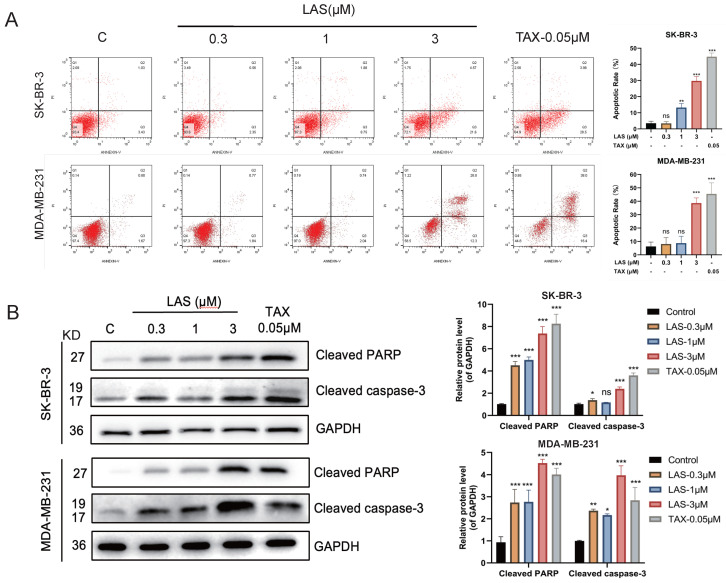
** LAS induced apoptosis of breast cancer cells. (**A)The apoptosis was detected by flow cytometry in SK-BR-3 and MDA-MB-231 cells treated with LAS for 48 h. (B) Detection of apoptosis marker proteins by Western Blot. Data are represented as the mean ± SD of three independent experiments. The *p*-values < 0.05 were considered statistically significant for all tests (*p < 0.05, **p< 0.01, ***p < 0.001).

The growth inhibition of cancer cells is regulated by a variety of factors, including inducing apoptosis, inducing cell cycle arrest, inhibiting signal transduction, and altering metabolic pathway 34. First, we tested whether LAS induces apoptosis of breast cancer cells by using Annexin V/PI double staining and immunoblotting assays. As shown in Fig. 2A, LAS treatment significantly increased the apoptotic cell rate of breast cancer cells SK-BR-3 and MDA-MB-231 in a concentration-dependent manner compared to that of controls. Moreover, immunoblotting results showed that LAS could significantly increase the protein levels of the apoptotic markers, including cleaved caspase-3 and cleaved PARP (Fig. 2B). Collectively, these results demonstrate that C118P induces apoptosis on breast cancer cells.

### LAS induced G2/M phase arrest of breast cancer cells

**Figure 3:**
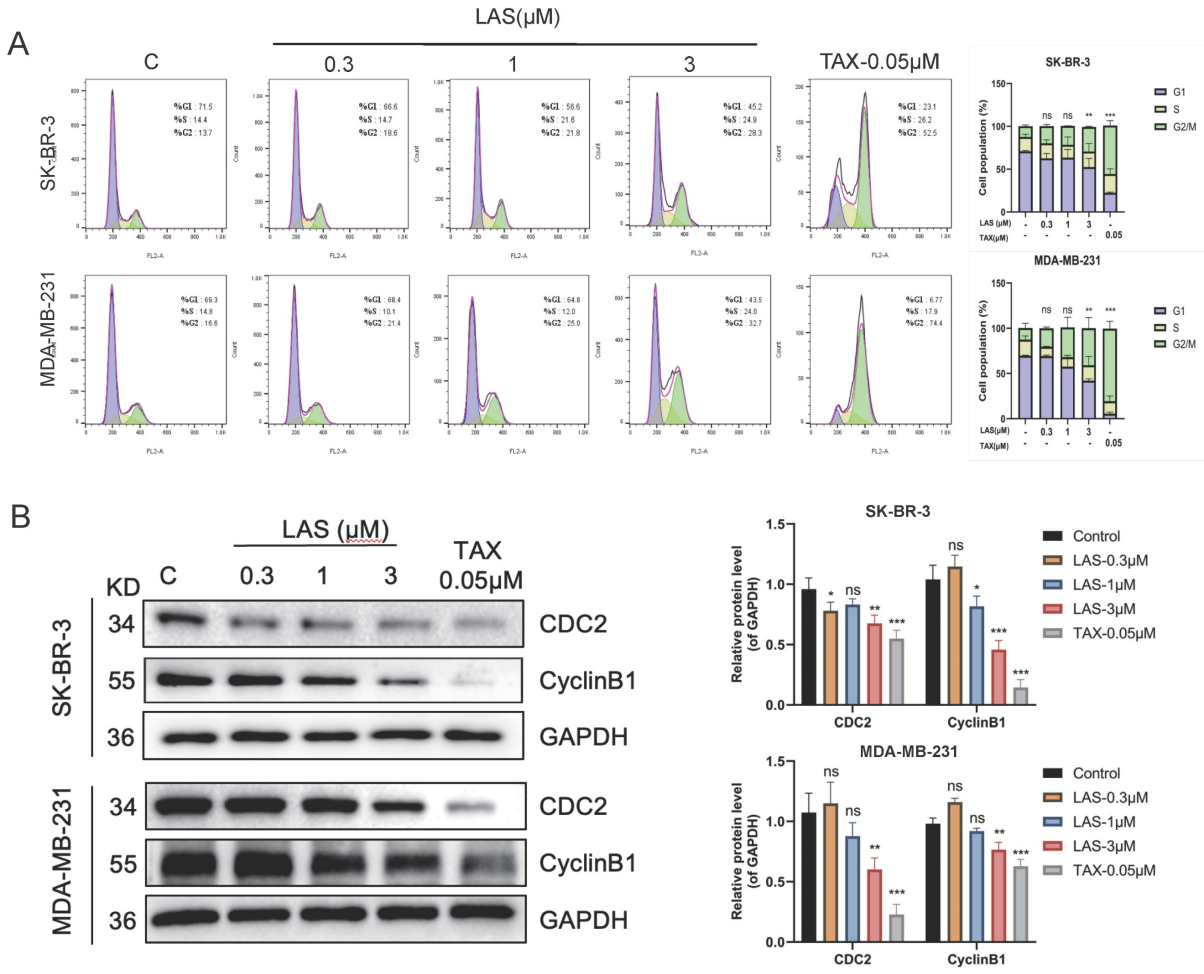
** LAS induced G2/M phase arrest of breast cancer cells.** (A) Cell cycle of MDA-MB-231 and SK-BR-3 treated with LAS for 48 h. PI staining assay was performed to detect the distribution of cell cycle. TAX was used as positive control. (B) Detection of G2/M phase arrest marker proteins by Western blot. Data are represented as the mean ± SD of three independent experiments. The p-values < 0.05 were considered statistically significant for all tests (*p < 0.05, **p< 0.01, ***p < 0.001).

To evaluate the effect of LAS on cell cycle of BC cell lines SK-BR-3 and MDA-MB-231, we performed flow cytometry assays (PI staining) and immunoblotting assays. As shown in Fig. 3A, flow cytometry showed that LAS (3 μM) could induce breast cancer cells in G2/M stage, with a significant increase in the proportion of cells in the G2/M phase in LAS treated SK-BR-3 and MDA-MB-231 compared to that of controls. Furthermore, our data showed that the protein expression of CDC2 and CyclinB1, the G2/M phase marker, were significantly decreased in BC cells SK-BR-3 and MDA-MB-231 with LAS treatment compared to that of controls (Fig. 3B). Thus, our findings suggest that LAS induces G2/M phase arrest in breast cancer cells.

### LAS induced G2/M phase arrest by suppressing PLK1 expression

**Figure 4:**
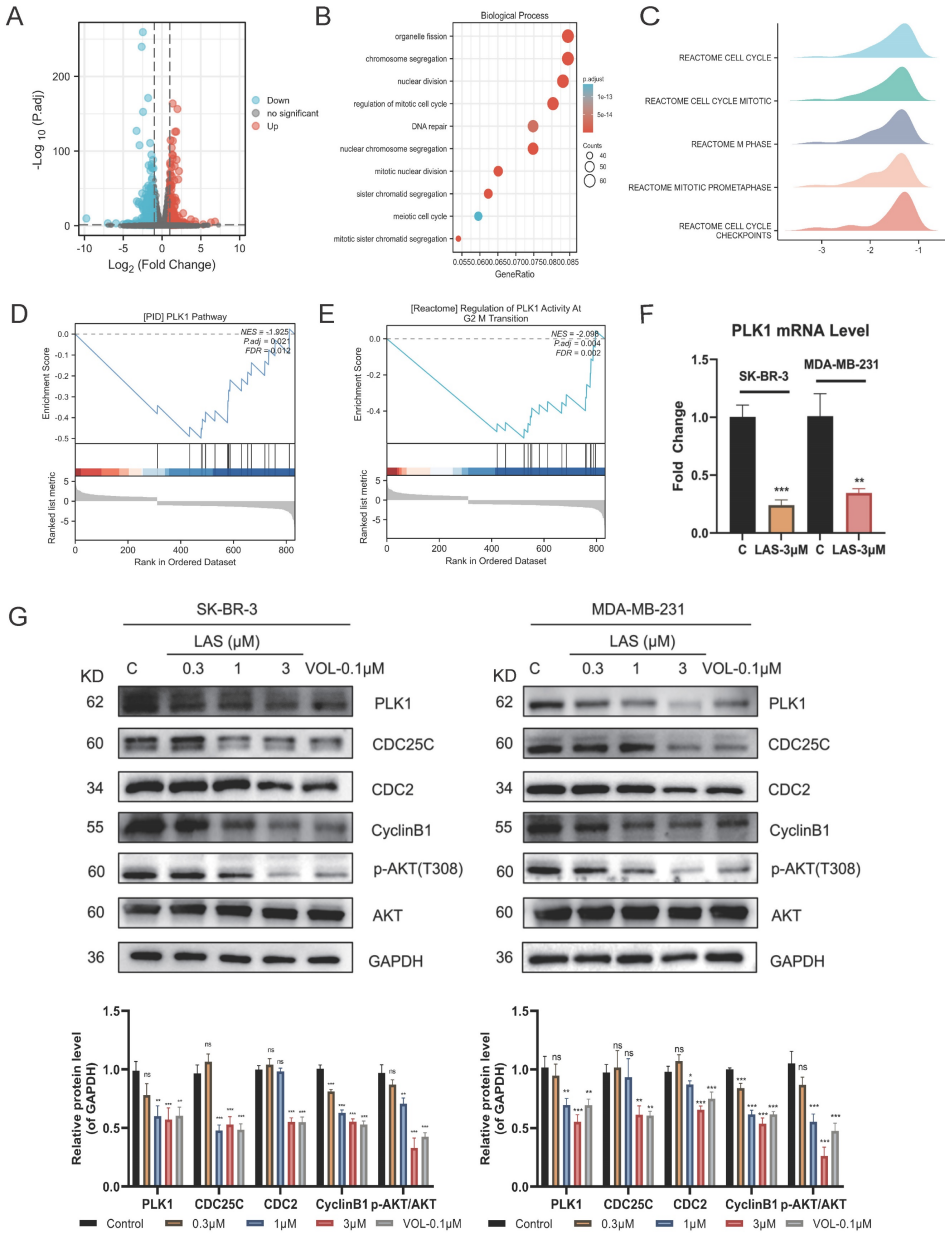
** LAS induced G2/M phase arrest suppressing PLK1 expression.** (A) Volcano Plot of differentially expressed genes. The blue ones are genes that are down-regulated, the red ones are up-regulated. (B) GO enrichment analysis of differentially expressed genes. (C-E) Gene Set Enrichment Analysis of differentially expressed genes. (F) PLK1 mRNA levels in breast cancer cells after LAS treatment. (G) Effect of LAS on PLK1 and downstream protein expression. Data are represented as the mean ± SD of three independent experiments. The *p*-values < 0.05 were considered statistically significant for all tests (*p < 0.05, **p< 0.01, ***p < 0.001).

To explore the mechanism by which LAS exerts anti-tumor effects on breast cancer, we conducted transcriptomic sequencing of SK-BR-3 cells with or without LAS treatment. Volcano plot showed that the expression of 916 genes were significantly changed in BC cells treated with LAS compared to that of controls (*p*<0.05), of which 330 genes were up-regulated and 586 genes were down-regulated (Fig. 4A). GO pathway enrichment analysis was performed on all genes with significant changes, and it was found that the top ten pathways with the most significant changes were related to the cell cycle, which was also consistent with the previous experimental results (Fig. 4B). Gene set enrichment analysis (GSEA) by analyzing all the significantly changed genes revealed that, in line with our experiment results that LAS influenced the cell cycle progression of breast cancer, the top five gene sets obtained all pointed to the mitotic phase of the cell (Fig. 4C). Taken together, our findings suggest that LAS exerts anti-tumor effects mainly by inducing G2/M phase arrest of breast cancer cells.

Further data from GSEA indicated that PLK1, play an important role in regulating the process of cell cycle, was a potential target of LAS (Fig. 4D-E). To verify the conjecture, we assessed the expression of PLK1 in BC cells using qRT-PCR and immunoblotting assays. As shown in Fig. 4F-G, LAS treatment significantly decreased the mRNA and protein levels of PLK1in both SK-BR-3 and MDA-MB-231 cells compared to that of controls. CDC25C, the downstream substrate of PLK1, could be activated by dephosphorylation of CDC2/CyclinB1 complex and participating in the regulation of G2/M phase of cells. In addition, PLK1 can also affect cell survival through the AKT pathway. We found that proteins expression of CDC25C and p-AKT(T308) were significantly decreased in SK-BR-3 and MDA-MB-231 cells treated with LAS (1,3 μM) compared to that of controls, suggesting that LAS inhibited the phosphorylation of CDC25C and AKT via regulating PLK1 pathway in breast cancer cells. Such effect of LAS comparable to a well-known kinase activity inhibitor of PLK1 named Volasertib (Fig. 4G). Taken together, these data suggest that LAS may induce G2/M phase arrest of breast cancer cells by regulating PLK1 expression.

### LAS inhibited breast cancer growth *in vivo*

**Figure 5:**
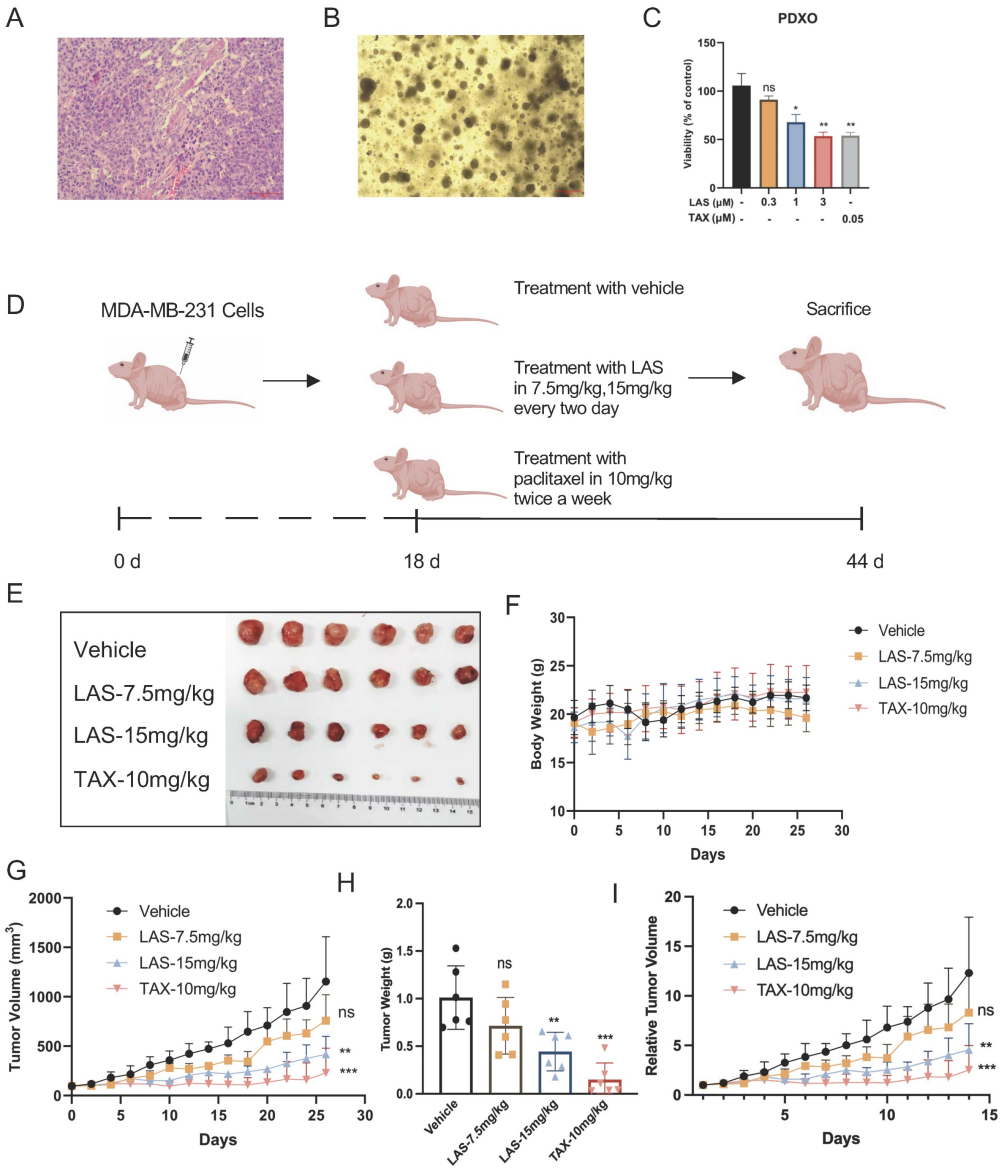
** LAS inhibited breast cancer growth *in vivo*.** (A-C) Effects of LAS on the proliferation of human breast cancer organoid. (A) Pathological section of PDX. (B) Bright-field images depicting major breast cancer organoid phenotypes. (C) CCK-8 assays showed that LAS suppressed organoid proliferation. (D) Experimental flow chart. (E) Schematic diagram of the tumor isolation from body. (F) Schematic diagram of tumor volume. (G) Schematic diagram of tumor weight. (H) Schematic diagram of body weight. (I) Schematic diagram of relative tumor volume.

We next established breast cancer organoids derived from patient-derived xenografts models (PDXO model) (Fig. 5A-B) to further confirm the anti-tumor activities of LAS on breast cancer. These organs are derived from tumors of clinical patients, which basically maintain the heterogeneity and organizational structure characteristics of tumors, and more completely reflect the actual situation of patients 35. By using this PDXO model, our findings demonstrated that LAS (1, 3μM) significantly inhibited cell viability on breast cancer organoids in a concentration-dependent manner (Fig. 5C), which not only verified the effect of LAS on breast cancer proliferation, but also provided a strong basis for the anti-tumor effect of LAS *in vivo*.

To verify the anti-tumor effect of LAS on breast cancer *in vivo*, we constructed a MDA-MB-231 xenograft mouse model (Fig. 5D). As shown in Figs. 5E-I, LAS inhibited the tumor growth in tumor bearing nude mice compared to that of controls. The tumor volume and tumor weight were significantly smaller and lower in the LAS (15 mg/kg) treated mice than vehicle treated group. Moreover, the body weight of LAS treated mice did not decrease significantly compared to that of negative controls, indicating no obvious toxicity of LAS (Figure 5F).

**Figure 6:**
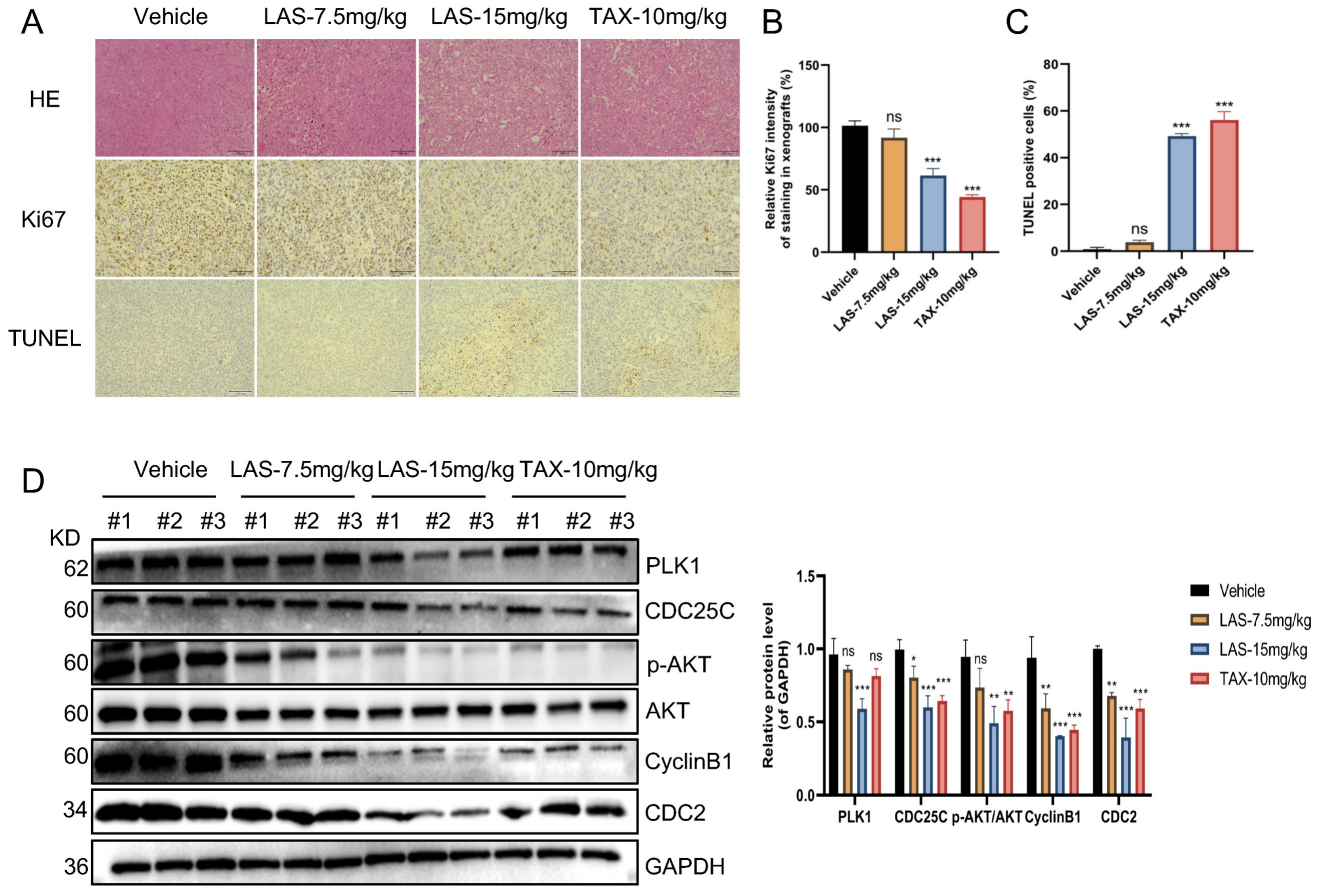
** LAS inhibited breast cancer growth by suppressing PLK1 expression *in vivo*.** (A) Representative images of H&E stained and immunohistochemical stained sections of MDA- MB-231 subcutaneous tumors after LAS or TAX treatment. scale bar: 100μm. (B) Bar graph of Ki-67 positive rate of MDA-MB-231 subcutaneous tumors. (C) Bar graph of TUNEL positive rate of breast tumors. (D) The expression levels of PLK1、CDC25C、 CyclinB1、CDC2 and p-AKT were all downregulated in tumor tissues. Three tumor tissues in each group were randomly selected for the experiment. The *p*-values < 0.05 were considered statistically significant for all tests (*p < 0.05, **p< 0.01, ***p < 0.001).

Furthermore, immunohistochemical staining assays showed that Ki67 expression was significantly decreased in tumor tissue of LAS treated tumor bearing nude mice compared to that of negative controls, while TUNEL positive cells markedly increased, suggesting that LAS can significantly inhibit the BC cells proliferation and induce the apoptosis of breast cancer cells *in vivo* (Fig.6A-C). In addition, the effects of LAS on PLK1-CDC25C and PLK1-AKT pathway *in vivo* were verified by immunoblotting assays. As shown in Fig.6D, the protein levels of PLK1, CDC25C, CyclinB1, CDC2 and p-AKT/AKT were significantly decreased in tumor tissue of LAS treated group compared to that of negative controls, which was consistent with the data shown in Fig. 4G. Taken together, our data demonstrate that LAS exerts its anti-tumor effect against breast cancer by suppressing PLK1 signaling.

## Discussion

*Isodon rubescens* has been employed as an adjuvant medicine for treatment of tumors 36, 37. The majority of reports on the bioactive components of Isodon rubescens focus on oridonin, with few studies on others. LAS, one of the main active ingredients, was first reported in 1975 and more recently studied as an intermediary in the synthetic process 38. Here, we first reported that LAS exerts anti-tumor effect against breast cancer. In our study, results from multiple research methods illustrate that LAS induces cellular G2/M blockade to trigger apoptosis via downregulating the PLK1 pathway, which suggests LAS could be a promising therapeutic agent for treatment of breast cancer.

Lasiokaurin, an ent-kaurane diterpenoid derived from *Isodon rubescens*, has recently been shown to have anti-cancer activity 14. Our current studies both *in vitro* and *in vivo* demonstrated that LAS remarkably inhibits the growth of breast cancer. Importantly, this anti-proliferatory effect of LAS was also verified by using PDXO model that maintain genetic heterogeneity of the original tumor and have higher clinical relevance 39.

Consistent with our experiment data that LAS significantly induced breast cancer cell cycle arrest in G2/M phase, GO enrichment analysis ranked "organelle fission", "chromosome segregation", "nuclear division", "regulation of mitotic cell cycle", and "DNA repair" etc. as the top ten biological processes with the most significant changes, which are all related to the cell cycle 40. Previous studies suggested that PLK1 contributes to the regulation of cell cycle progression and DNA replication 41. Moreover, extensive studies have revealed that PLK1 was overexpressed in a variety of human cancers and associated with poor prognoses in multiple types of human cancers 42. Our findings here by using RNA sequencing and biochemistry assays demonstrated that LAS suppressed the expression of PLK1, accompanied by inhibiting AKT pathway and CDC25C pathway that reported as its downstream pathways involved in the process of cell cycle and cell survival.

## Conclusion

In summary, LAS can effectively inhibit breast cancer growth via inducing cell cycle arrest and apoptosis both *in vivo* and *in vitro*. This study preliminarily predicted the mechanism of LAS by RNA sequencing analysis, which was then further verified by experiments. Our findings here demonstrated that LAS exerts anti-tumor effects by inhibiting PLK1/CDC25C and PLK1/AKT pathways. PLK1, a potential target for cancer therapy, was revealed for the first time to regulated by LAS in breast cancer. However, how LAS affects PLK1 expression and whether LAS exerts its anti-cancer activities via regulating other pathways, or shares imilar molecular mechanisms with *Isodon rubescens* are worthy of further investigations.
